# Definition of a coordinate system for multi-modal images of the temporal bone and inner ear

**DOI:** 10.1371/journal.pone.0294828

**Published:** 2024-10-07

**Authors:** Bridget Copson, Sudanthi Wijewickrema, Christopher Slinger, Daniel Youssef, Jean-Marc Gerard, Stephen O’Leary

**Affiliations:** 1 Department of Otolaryngology (Surgery), University of Melbourne, Melbourne, Australia; 2 Department of Radiology, St Vincent’s Hospital, Melbourne, Australia; 3 Department of Otology, Royal Victorian Eye and Ear Hospital, Melbourne, Australia; Ohio State University, UNITED STATES OF AMERICA

## Abstract

**Purpose:**

The position and orientation of the head is maintained to be relatively similar during the CT / MR imaging process. However, the position / orientation dissimilarities present in the resulting images between patients, or between different scans of the same patient, do not allow for direct comparison of the images themselves or features / metrics extracted from them. This paper introduces a method of defining a coordinate system which is consistent between patients and modalities (CT and MR) for images of the temporal bone, using easily identifiable landmarks within the semicircular canals.

**Methods:**

Cone Beam CT and high resolution MRI (T2) images of the temporal bone from 20 patients with no cochlear or temporal bone pathology in either modality were obtained. Four landmarks within the semicircular canals were defined that can be identified in both modalities. A coordinate system was defined using these landmarks. Reproducibility of landmark selection was assessed using intra- and inter-rater reliability (for three expert raters and two repeats of the landmark selection). Accuracy of the coordinate system was determined by comparing the coordinates of two additional landmarks in CT and MR images after their conversion to the proposed coordinate system.

**Results:**

Intraclass Correlation Coefficients at a 95% level of confidence showed significant agreement within and between raters as well as between modalities. The differences between selections, raters, and modalities (as measured using mean, standard deviation, and maximum) were low and acceptable for clinical applications.

**Conclusion:**

The proposed coordinate system is suited for use in images of the temporal bone and inner ear. Its multi-modal nature enables the coordinate system to be used in tasks such as image co-registration.

## Introduction

Computed Tomography (CT) and Magnetic Resonance Imaging (MRI) are two frequently performed cross section imaging modalities used for temporal bone imaging for the purposes of diagnosing otological and cerebellopontine angle (CPA) disorders, in addition to surgical planning and intra-operative imaging guidance. Each modality serves a particular benefit: CT informs an accurate assessment of osseous landmarks of the petrous temporal bone and MR images support the understanding of soft tissue and fluid filled structures such as the membranous labyrinth and the internal acoustic meatus (IAM). While these images are acquired using vastly different imaging techniques, the practicalities of imaging are such that the position of the patient’s head is maintained to be relatively similar in each individual scan. However, given the fine structures that are imaged in the temporal bone, even small discrepancies in orientation and position cause unique difficulties when attempting to directly compare images themselves or the metrics extracted from them. Creating a reference orientation / position (or a common coordinate system) which all scans could be aligned upon for direct comparison remains a challenge.

The benefit of such a coordinate system in lateral skull base imaging and surgery would be multi-fold. Firstly, a coordinate system would allow for surgical planning across two modalities, obtaining the detail of the osseous and membranous labyrinths in a single set of coordinates. This data could also be used to assess and identify anatomic variation, structural dysplasias, and pathology.

There are various landmarks and planes which have been used for the purpose of defining a coordinate system of the base of skull and facial bones, with many studies performed for the purpose of maxillofacial surgery [1–4]. Commonly used planes include the Frankfurt horizontal plane and the Reid horizontal plane. These planes rely on imaging of the orbits, and other commonly used landmarks rely on imaging of the maxilla and mandible, none of which are consistently visualised on high resolution CT and MRI used for temporal bone / inner ear imaging. For the purposes of image guided surgery, a commonly used and accurate option is the use of an external fiducial system, where imaging is acquired with externally fixated markers. While these markers have been shown to be accurate for the use in temporal bone surgery [5], they are invasive [6] and add time to the surgery and require application prior to imaging acquisition [7]. In addition, the fiducial system means that a coordinate system cannot be applied retrospectively. As such, a coordinate system more suited for temporal bone / inner ear surgery, based landmarks such as the semi-circular canals should be defined.

Establishment of a coordinate system requires an origin and axes / planes normal to each other to be defined so that Cartesian coordinates can be used. Multiple systems have been developed by defining axial and sagittal planes using specific landmarks, whilst coronal planes are often extracted from these. Landmarks, for the purpose of constructing coordinate systems, have been defined using patient scans themselves [8, 9] or 3D reconstructions derived from these [10–12]. High degrees of reliability and reproducibility are required in landmarks that define such a coordinate system. While reliability ensures that the chosen landmarks are visible and identifiable between patients, ideally even in the presence of malformations, reproducibility defines the ease with which different experts can identify the landmarks consistently. In the case of multi-modal coordinate systems, these characteristics have to be applicable for different imaging modalities such as CT and MR as well.

Although the morphology of the semi-circular canals has long been studied [13, 14], a comprehensive understanding of the three dimensional morphology has only more recently been demonstrated [15–17]. While some of these studies have found that the semi-circular canals are not perfectly planar [17, 18], they are often approximated as such for practical purposes. Furthermore, Santina et al. [15] provides significant evidence as to the orientation of the semi-circular canals with respect to each other and the Reid coordinate system [19] using CT scans of 22 subjects. They show that the angles between the left and right lateral / horizontal semi-circular canals and the Reid horizontal plane was 19.5^*o*^±7.4^*o*^ and 20.4^*o*^±6.9^*o*^ respectively. They also found no significant difference between the centers of each pair of semi-circular canals (left and right) and the Reid stereotaxic planes. The left and right lateral semi-circular canals (LSCCs) lie roughly on the same plane, as indicated by the fact that they form a plane roughly ∼20^*o*^ from the Reid horizontal plane, and symmetrical points on the left and right LSCCs are equidistant from this plane.

The plane of the lateral semicircular canals has been previously used as a reference system in the setting of craniofacial surgery in the setting of base of skull asymmetry due to pathology, where other landmark systems are inappropriate due to their reliance on calvarial symmetry [20]. This plane is acquired by defining the centre-point of the lateral semicircular canal (LSCC) and a second axis traversing the anterior and posterior limits of the LSCC [20]. There have been a few further studies using this plane, predominantly used in the setting of facial asymmetry [21], and with the additional landmark of the nasion (nasal bridge) for orthognathic surgery [8]. Both these studies demonstrated this plane to be reproducible and comparable to other commonly used landmark-oriented reference planes.

In this study, we assessed the application of a modified lateral semi-circular canal plane to cone beam CT and high resolution MRI of the temporal bone. The hypothesis of this study was that by choosing specific and reliable landmarks related only to the lateral semicircular canal which can be seen in both cone beam CT and high resolution MRI (without additional landmarks of the facial bones), a reproducible and accurate coordinate system can be obtained. An added byproduct of choosing landmarks visualised in both modalities is that an accurate, automated co-registered cone beam CT and MRI image can be processed.

## Materials and methods

### Definition of the coordinate system

We used the lateral semi-circular canals (LSCCs) as an intuitively appropriate position on which to base the proposed coordinate system, as there are only few landmark options visualised consistently on both CT and MRI. The LSCC is a common orientating landmark used during cochlear implant surgery, where the surgeon relies on an excellent mental representation of the patient’s anatomy during electrode placement [22]. Furthermore, these structures allow two regions of interest (ROI) to be plotted, helping to minimise bias of asymmetry [20]. The specific points of the anterior and posterior portion of the LSCCs were chosen as reliably identified landmarks clearly and consistently visible in both CT and MR images.

**Fig 1:**
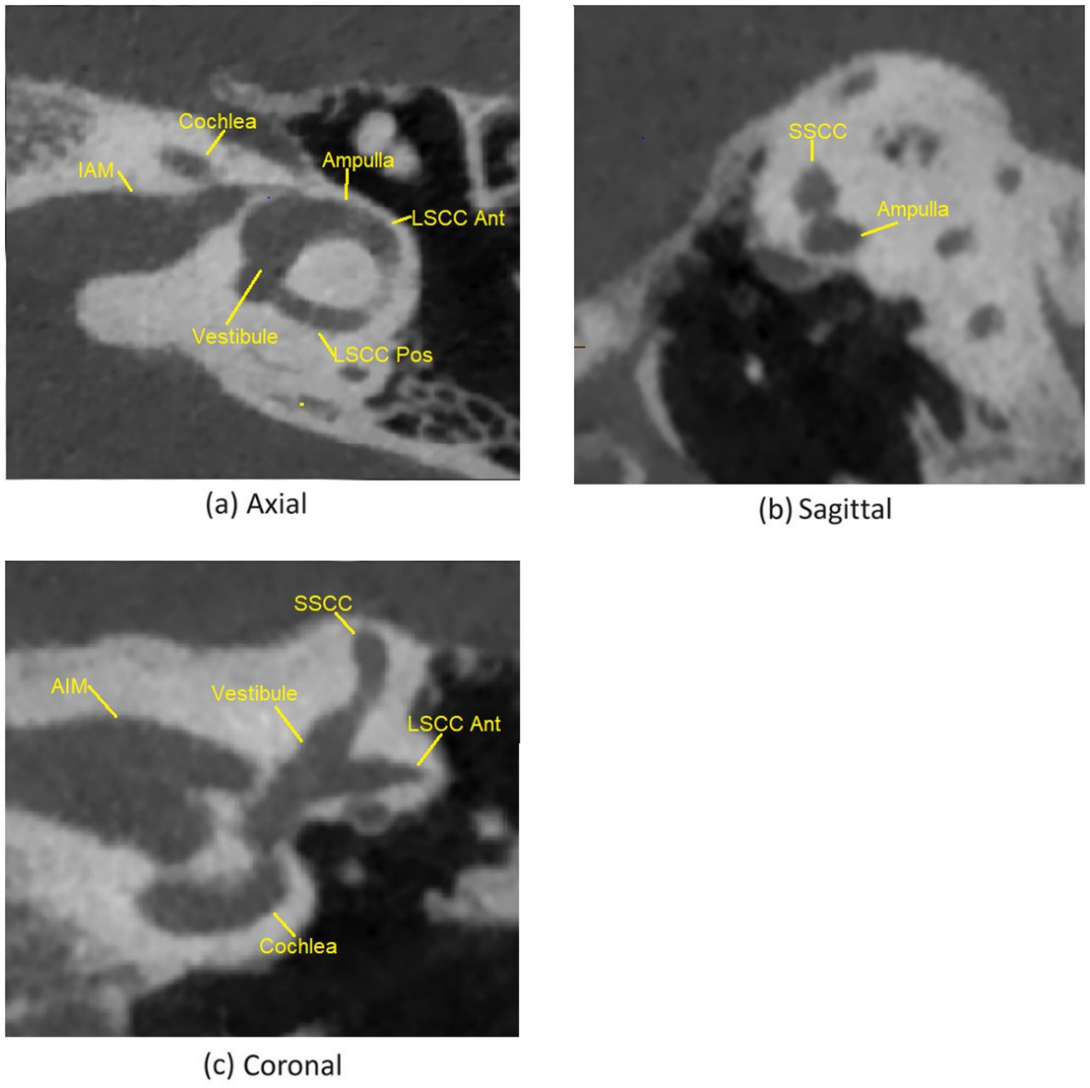
The CT view of the relevant anatomy for the proposed landmarks. IAM: internal auditory meatus / internal auditory canal. LSCC ant: the anterior aspect of the lateral semicircular canal, the anterior aspect of which is the ampulla. LSCC Post: posterior aspect of the lateral semicircular canal. Vestibule, Cochlea.

**Fig 2:**
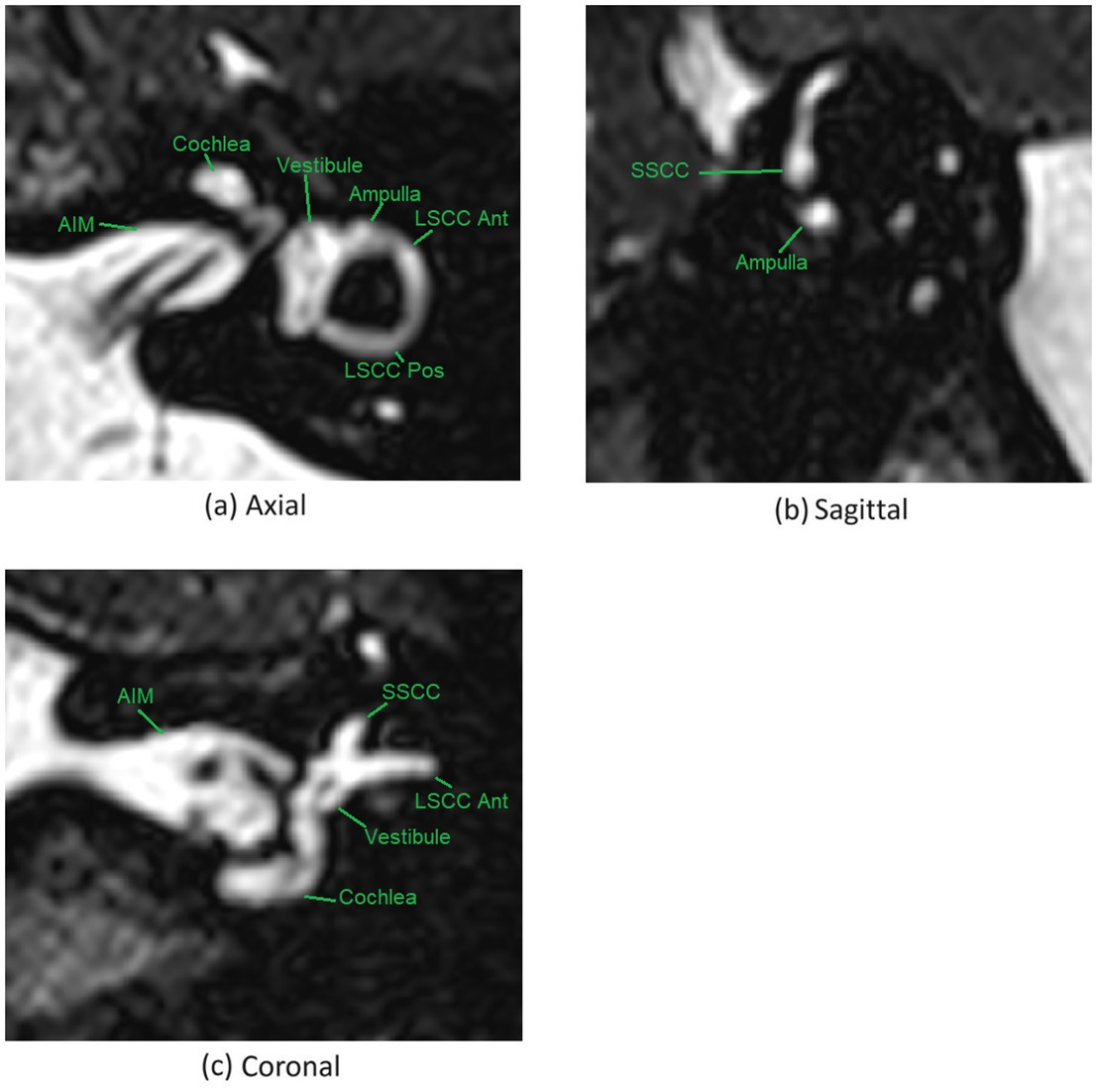
The relevant anatomy for the proposed landmarks as viewed in MR. IAM: internal auditory meatus/ internal auditory canal. LSCC ant: the anterior aspect of the lateral semicircular canal, the anterior aspect of which is the ampulla. LSCC Post: posterior aspect of the lateral semicircular canal. Vestibule, Cochlea.

We used two symmetric points (anterior and posterior) on the lateral semi-circular canals as the basis for defining our proposed coordinate system. The anterior landmark was defined as the junction between the anterior aspect of the LSCC (within the ampulla) and the vestibule, at the centre-point of this junction as viewed in the three planes. The LSCC was identified on the sagittal image, and then followed anteriorly to the ampulla, until the point of confluence with the vestibule was identified and the centre-point of the LSCC at this location was confirmed in all three planes. The posterior landmark was defined as the junction between the posterior aspect of the LSCC and the vestibule, at the centre-point of this junction as viewed in the three planes. The LSCC was followed posteriorly until the point of confluence with the vestibule was identified and the centre-point of the LSCC at this location was confirmed in all three planes (posterior landmark). CT and MR views of the anatomy related to the landmark selection are shown in Figs 1 and 2 respectively.

**Fig 3:**
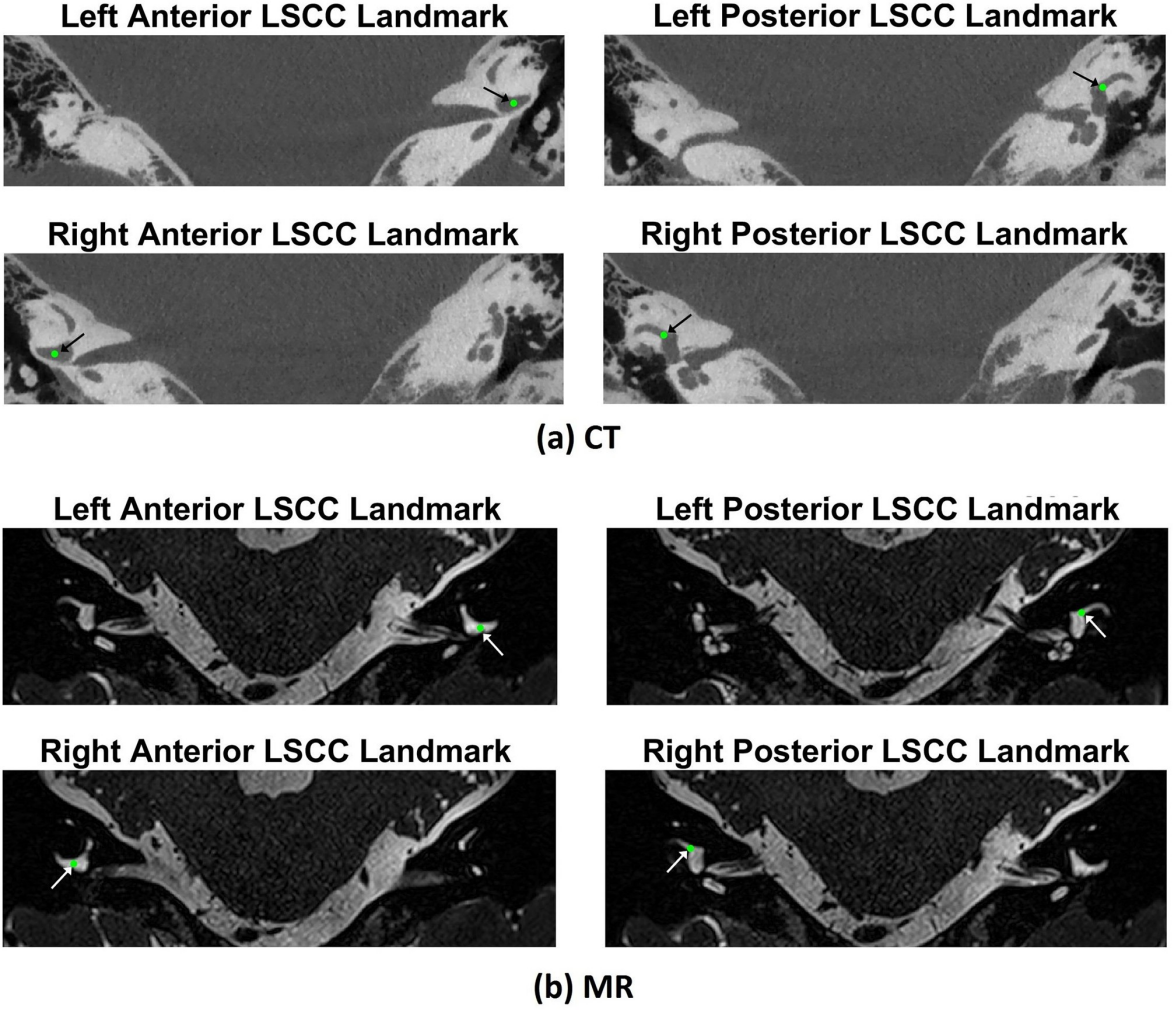
Lateral semi-circular canal (LSSC) landmarks selected from axial slices of the image stacks: (a) CT and (b) MR of the same patient. The landmarks are shown as green dots. The images have been cropped around the region of interest for clarity. The coordinates were obtained from a right handed system where the axes take the following directions. X: right to left, Y: anterior to posterior, and Z: inferior to superior. The four images of each image modality correspond to the axial slices at the Z coordinates of the selected points.

We named the above points as: ‘Left Anterior LSCC’, ‘Left Posterior LSCC’, ‘Right Anterior LSCC’ and ‘Right Posterior LSCC’. These landmarks were chosen as they form the basis of the current multi-planar reformats used in our institutions for the volumetric reconstruction of cone beam CTs and because they can be easily detected in both MR and CT scans. Fig 3 shows an example of how these landmarks are identified in CT and MR scans.

Next, we fit a plane (using least-squares fitting) to these four landmarks to represent the plane of the LSCC. Due to human error, the discrete nature of the the coordinates in a scan, and the fact that the LSCCs may not be perfectly planar, the landmarks may not fall perfectly on a plane. As such, we calculated the orthogonal projections of these landmarks on the plane, and used these projections in the definition of our coordinate system. We defined the origin of the system as the center (mean) of the four projected points. The X axis was defined to go through the mid points of the lines joining the anterior and posterior points on each side. The direction of the x axis was from right to left. We defined the y axis as the normal to the x axis in the anterior-posterior direction on the LSCC plane. Then, the z axis was defined as the vector normal to the xy plane (LSCC plane) that formed a right handed coordinate system with the x and y axes and was in the inferior-superior direction.

**Fig 4:**
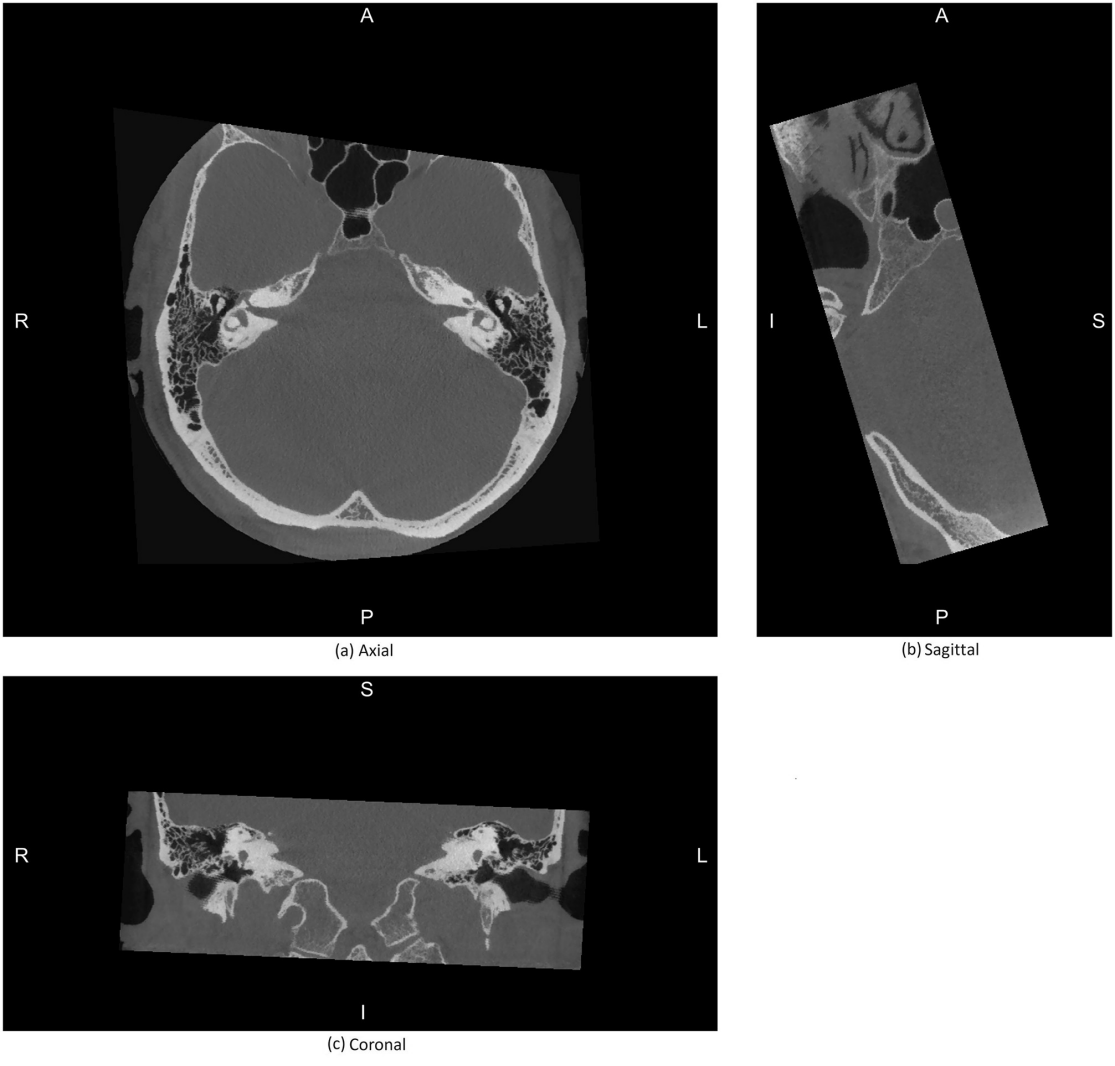
Mid slices of a CT image after it has been transformed to the proposed coordinate system. A, P, S, I, R, and L indicate anterior, posterior, superior, inferior, right, and left directions respectively.

**Fig 5:**
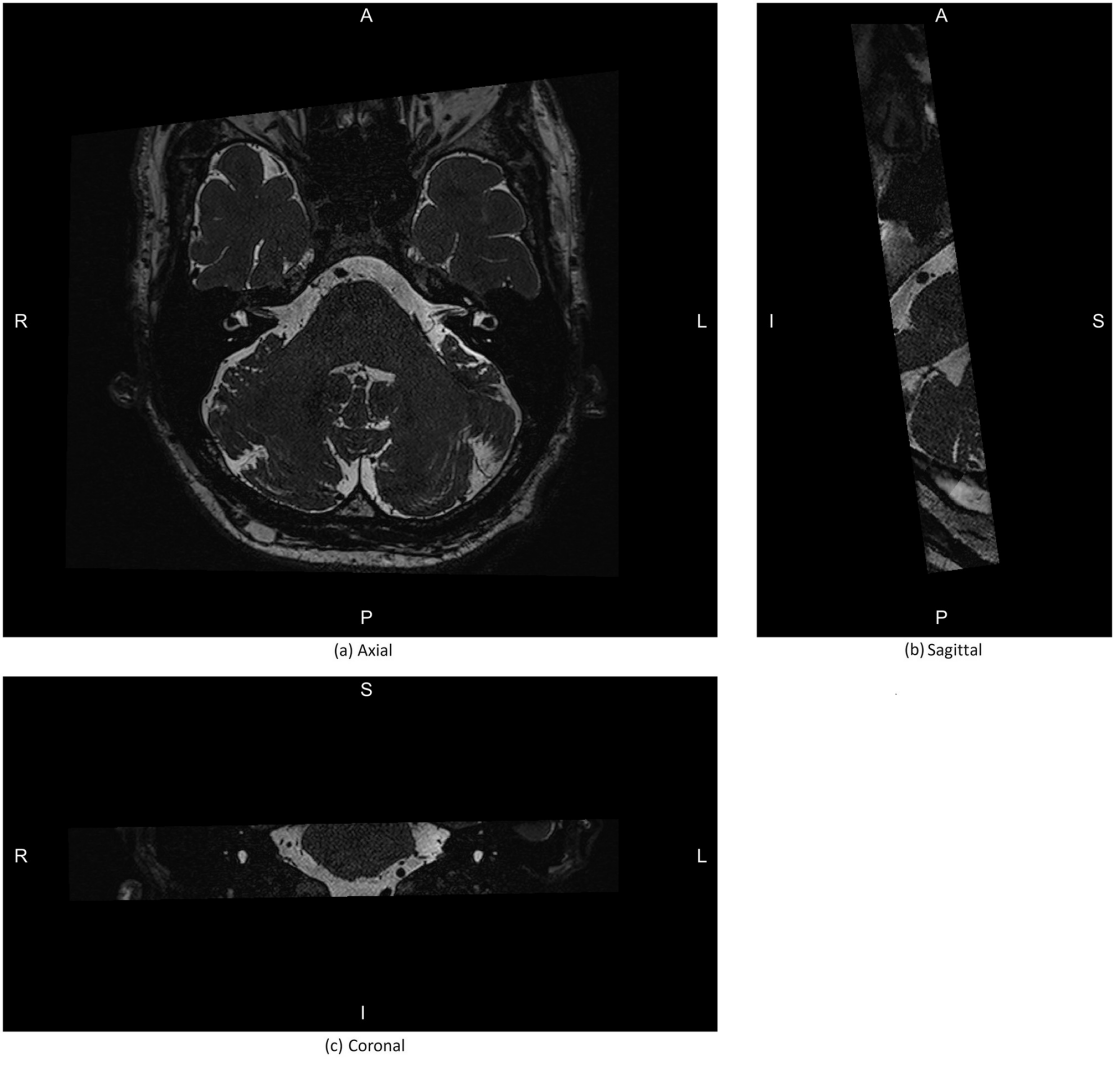
Mid slices of a MR image in the proposed coordinate system. A, P, S, I, R, and L indicate anterior, posterior, superior, inferior, right, and left directions respectively.

Although this coordinate system can be consistently defined across patients and modalities using internal landmarks, it varies considerably from the more familiar upright orientation of the head. To address this, we rotated our coordinate system by 20° counter-clockwise around the x axis so that the xy (LSCC) plane was closer to the Reid horizontal plane. However, note that the aim of this work was not to define a coordinate system aligned with the true horizontal plane, and as such, this rotation was performed only for the purpose of convenience in visualisation. Figs 4 and 5 show the mid slices after the CT and MR volumes have been transformed into the proposed coordinate system.

### Data

We obtained Cone Beam CT and MRI (T2) data from 20 patients over a 3 year period (2016 -2019), with no cochlear or temporal bone pathology in either MRI or cone beam CT as assessed by the reporting radiologist. Patients with CT and MR scans of resolutions of [0.15, 0.15, 0.15] and [0.2604, 0.2604, 0.3] mm per voxel respectively and at least 300 and 80 slices in the axial direction respectively were included. Ethics approval for data collection for this project was obtained from the Royal Victorian Eye and Ear Hospital Human Ethics Committee (#08–796H-13). The data was accessed for research purposes from August 2022 to May 2023. The data was fully anonymised before it was used in the study and the requirement for informed consent was waived by the Ethics committee.

### Experimental setup

We used the software ITK-SNAP to identify landmarks for the determination of the coordinate system. We used MATLAB to implement methods and conduct statistical tests. We used a significance level of 95% in all statistical tests.

### Reproducibility

We asked three different experts: two Otolaryngologists and a Radiologist (7, 22 and 6 years of experience respectively), to select the landmarks defined above, twice each for the MR and CT images.

To investigate the reproducibility in choosing landmarks, we tested for intra- and inter-rater reliability. We compared X, Y, and Z coordinates of the different landmarks separately due to their independence. We used the intra-class correlation coefficient (ICC) [23, 24] as the statistical test because it tests the reliability, the extent to which measurements can be replicated, reflecting the degree of correlation as well as the agreement between measurements [25–27]. Out of the different calculations of ICC available [23], we chose a 2-way random effects model for multiple raters with absolute agreement as the most appropriate for our analyses [28]. We also determined the mean, standard deviation, and maximum of the differences in landmark coordinates [29]. When determining the intra-rater reliability, we calculated the above metrics for each rater separately. When calculating the inter-rater reliability, we used the mean coordinates from the 2 attempts by each rater. We compared the coordinates selected by each pair of raters separately and reported the worst results of the 3 combinations.

### Evaluation of accuracy

Transformation of images of different modalities into the same coordinate system effectively results in co-registration of the images. As such, the accuracy of the process can be determined using the target registration error (TRE), typically defined as the distance between landmarks in the registered images. The landmarks used in this calculation should not be those used in calculating the transformation parameters in the registration process [30, 31]. Here, we used the mean, standard deviation, and maximum of the Eucledian distance as the TRE [29]. The landmarks used were the mid point of the superior semicircular canals on each side, at their vertex. These landmarks were used as they were visible on both CT and MRI, and distant to the previously used landmarks. Note that the median coordinates of the original landmarks were used for the determination of the coordinate system in order to avoid bias and errors.

The superior semicircular canal (SSCC) landmarks were detected by one of the experts that chose the original landmarks. Prior to this, both the CT and MR images were transformed to the proposed coordinate system with the origin being aligned at the center of the image. All images were scaled to the voxel dimension of 0.2604mm in each direction.

Balnd-Altman plots [32] were used to visualise the difference between the co-registered CT and MR coordinates. Bland-Altman plots, also known as difference plots, are a convenient way to assess the agreement between two sets of data [33]. The y axis shows the difference between the two paired measurements and the x axis represents the average of these measures. Mean and 95% confidence intervals of the differences are also plotted. An ideal agreement is zero difference [34].

### Validation of the planarity assumption

As mentioned above, the left and right LSCCs lie (roughly) on the same plane. Since our coordinate system is based on this, it is prudent to test this assumption of planarity. For this, we first determine the median of the landmark coordinates selected by the experts. Then, we calculate the orthogonal projections of these points on the best-fit plane determined using them. These projected points are the points on the plane closest to the selected landmarks. Comparing the coordinates of the projected points and the original landmarks provides us with an idea of how close the landmarks are to being planar.

## Results

**Fig 6:**
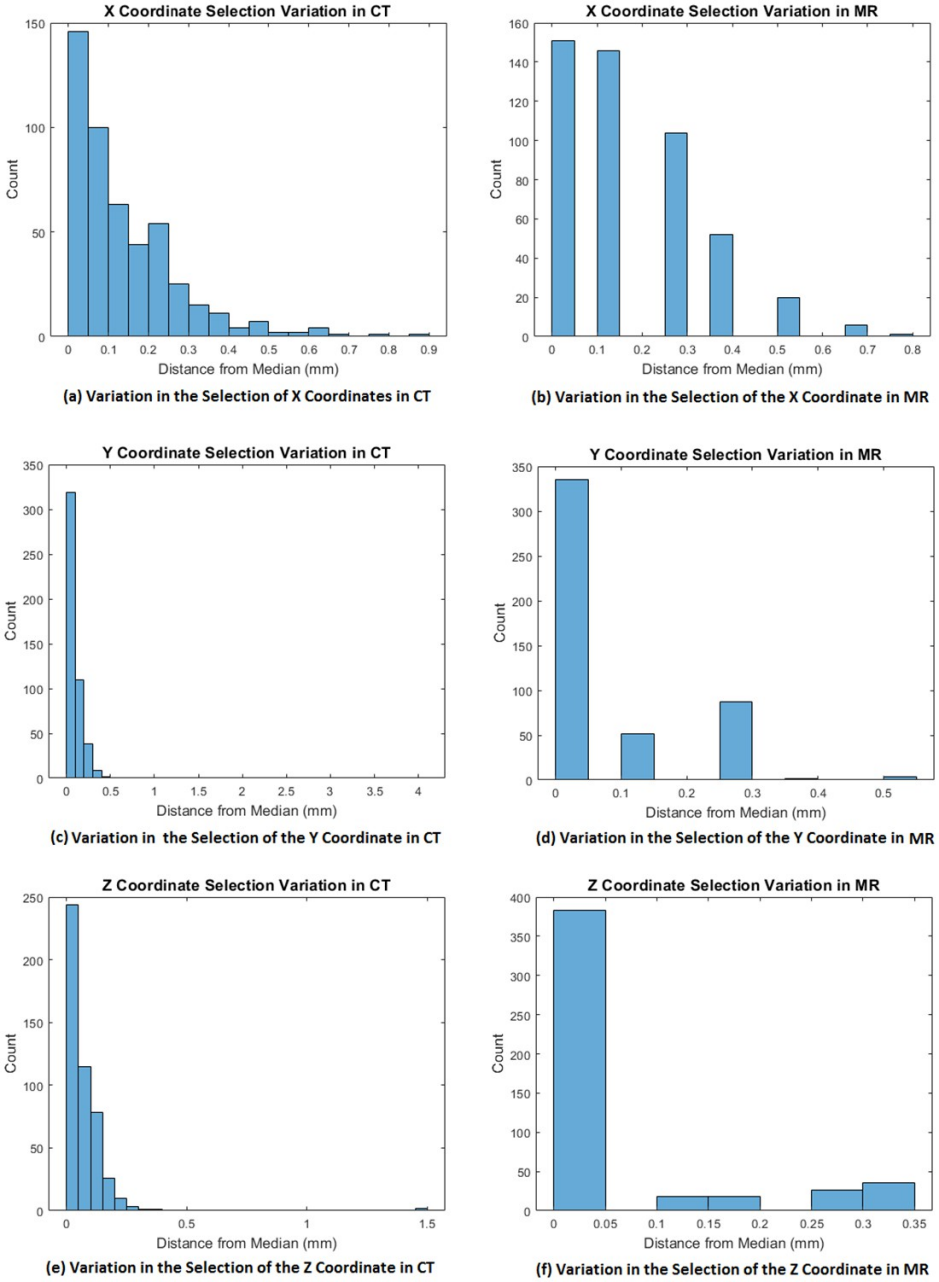
Difference of each selected landmark coordinate against the median of the 6 coordinate values selected by the 3 experts in 2 repetitions.

The results for intra-rater reliability are shown in Tables 1 and 2. Tables 3 and 4 show the inter-rater reliability for CT and MR respectively. The degree of absolute agreement between the coordinates (given by *r*) is high for all landmark coordinates and the results are statistically significant. The differences in mm are within acceptable levels. Fig 6 shows the variation in the landmark selection, calculated as the difference of each selected coordinate and the median of the 6 coordinate values (3 experts × 2 repetitions) for each point. Note that the majority of the differences are small.

**Table 1:** Intra-rater reliability for the 3 experts when selecting the proposed LSCC landmarks in CT. The voxel size in the X, Y, and Z directions is 0.15mm.

Landmark	Coordinate	r	p	Difference (mm)		
				Mean	Std Dev	Max
**Expert A**						
Left Anterior	X	0.9982	<0.001	0.1725	0.1839	0.6000
	Y	0.9998	<0.001	0.1500	0.1192	0.4500
	Z	0.9995	<0.001	0.1275	0.1313	0.4500
Left Posterior	X	0.9984	<0.001	0.1800	0.1727	0.6000
	Y	0.9998	<0.001	0.1650	0.1182	0.4500
	Z	0.9997	<0.001	0.0975	0.1006	0.3000
Right Anterior	X	0.9980	<0.001	0.1800	0.1983	0.7500
	Y	0.9999	<0.001	0.1425	0.0907	0.3000
	Z	0.9994	<0.001	0.1575	0.1139	0.4500
Right Posterior	X	0.9987	<0.001	0.1650	0.1368	0.4500
	Y	0.9994	<0.001	0.2175	0.2857	1.2000
	Z	0.9995	<0.001	0.0975	0.1313	0.3000
**Expert B**						
Left Anterior	X	0.9990	<0.001	0.1350	0.1278	0.4500
	Y	0.9999	<0.001	0.1050	0.0857	0.3000
	Z	0.9999	<0.001	0.0675	0.0766	0.1500
Left Posterior	X	0.9991	<0.001	0.1350	0.1182	0.4500
	Y	0.9999	<0.001	0.0825	0.1029	0.3000
	Z	0.9999	<0.001	0.0525	0.0734	0.1500
Right Anterior	X	0.9996	<0.001	0.0825	0.0907	0.3000
	Y	1.0000	<0.001	0.0525	0.0734	0.1500
	Z	0.9999	<0.001	0.0525	0.0734	0.1500
Right Posterior	X	0.9988	<0.001	0.1500	0.1376	0.4500
	Y	0.9999	<0.001	0.0900	0.1131	0.4500
	Z	0.9998	<0.001	0.0675	0.0766	0.1500
**Expert C**						
Left Anterior	X	0.9989	<0.001	0.1500	0.1288	0.3000
	Y	0.9999	<0.001	0.0900	0.0897	0.3000
	Z	0.9999	<0.001	0.0600	0.0754	0.1500
Left Posterior	X	0.9982	<0.001	0.1725	0.1963	0.9000
	Y	0.9956	<0.001	0.3075	0.8860	4.0500
	Z	0.9965	<0.001	0.2250	0.4474	1.5000
Right Anterior	X	0.9987	<0.001	0.1650	0.1368	0.4500
	Y	0.9999	<0.001	0.1050	0.0985	0.3000
	Z	0.9998	<0.001	0.0675	0.0907	0.3000
Right Posterior	X	0.9990	<0.001	0.1350	0.1368	0.4500
	Y	0.9998	<0.001	0.1500	0.1288	0.4500
	Z	0.9998	<0.001	0.0600	0.0754	1.5000

**Table 2:** Intra-rater reliability and for the 3 experts for LSCC landmark selection in MR. The voxel dimensions in the X, Y, and Z directions are 0.2604mm, 0.2604mm, and 0.3mm respectively.

Landmark	Coordinate	r	p	Difference (mm)		
				Mean	Std Dev	Max
**Expert A**						
Left Anterior	X	0.9988	<0.001	0.1302	0.1581	0.5208
	Y	0.9999	<0.001	0.0391	0.0954	0.2604
	Z	0.9992	<0.001	0.0450	0.1099	0.3000
Left Posterior	X	0.9986	<0.001	0.1562	0.1558	0.5208
	Y	0.9997	<0.001	0.1432	0.1329	0.2604
	Z	0.9993	<0.001	0.0450	0.1099	0.3000
Right Anterior	X	0.9968	<0.001	0.2083	0.2618	1.0416
	Y	0.9999	<0.001	0.0651	0.1157	0.2604
	Z	0.9976	<0.001	0.1200	0.1795	0.6000
Right Posterior	X	0.9989	<0.001	0.1302	0.1581	0.5208
	Y	0.9995	<0.001	0.2083	0.1812	0.5208
	Z	0.9993	<0.001	0.0450	0.1099	0.3000
**Expert B**						
Left Anterior	X	0.9993	<0.001	0.0911	0.1274	0.2604
	Y	0.9998	<0.001	0.1042	0.1309	0.2604
	Z	0.9992	<0.001	0.0450	0.1099	0.3000
Left Posterior	X	0.9991	<0.001	0.0911	0.1529	0.5208
	Y	0.9999	<0.001	0.0521	0.1069	0.2604
	Z	0.9998	<0.001	0.0150	0.0671	0.3000
Right Anterior	X	0.9986	<0.001	0.1823	0.1224	0.2604
	Y	0.9999	<0.001	0.0651	0.1157	0.2604
	Z	0.9976	<0.001	0.1200	0.1795	0.6000
Right Posterior	X	0.9992	<0.001	0.1042	0.1309	0.2604
	Y	0.9999	<0.001	0.0651	0.1157	0.2604
	Z	0.9991	<0.001	0.0600	0.1231	0.3000
**Expert C**						
Left Anterior	X	0.9970	<0.001	0.2474	0.1977	0.7812
	Y	0.9999	<0.001	0.0391	0.1274	0.5208
	Z	0.9989	<0.001	0.0600	0.1231	0.3000
Left Posterior	X	0.9985	<0.001	0.1693	0.1529	0.5208
	Y	0.9996	<0.001	0.1562	0.1558	0.5208
	Z	0.9996	<0.001	0.0300	0.0923	0.3000
Right Anterior	X	0.9961	<0.001	0.2734	0.2310	0.7812
	Y	0.9999	<0.001	0.0651	0.1157	0.2604
	Z	0.9995	<0.001	0.0300	0.0923	0.3000
Right Posterior	X	0.9985	<0.001	0.1562	0.1772	0.5208
	Y	0.9997	<0.001	0.1693	0.1274	0.2604
	Z	0.9987	<0.001	0.0900	0.1410	0.3000

**Table 3:** Inter-rater reliability for experts in selecting the proposed LSCC landmarks in CT. The worst results from comparisons between each pair of raters are shown. The voxel size in each of the X, Y, and Z directions is 0.15mm.

Landmark	Coordinate	r	p	Difference (mm)		
				Mean	Std Dev	Max
Left Anterior	X	0.9907	0.0060	0.2775	0.1791	0.6000
	Y	0.9998	<0.001	0.1000	0.0765	0.3750
	Z	0.9997	<0.001	0.0600	0.0583	0.2250
Left Posterior	X	0.9996	<0.001	0.1825	0.1320	0.6000
	Y	0.9974	<0.001	0.1900	0.3568	2.1000
	Z	0.9974	<0.001	0.1250	0.1966	0.9000
Right Anterior	X	0.9925	0.0034	0.2350	0.1607	0.6750
	Y	0.9998	<0.001	0.0850	0.0762	0.3000
	Z	0.9994	<0.001	0.0850	0.0655	0.3000
Right Posterior	X	0.9981	<0.001	0.1200	0.0969	0.3750
	Y	0.9996	<0.001	0.1300	0.1179	0.6000
	Z	0.9996	<0.001	0.0700	0.0660	0.3000

**Table 4:** Inter-rater reliability when selecting LSCC landmarks in MR. The worst results from comparisons between each pair of raters are shown. The voxel dimensions are 0.2604mm, 0.2604mm, and 0.3mm in the X, Y, and Z directions respectively.

Landmark	Coordinate	r	p	Difference (mm)		
				Mean	Std Dev	Max
Left Anterior	X	0.9847	0.0101	0.3559	0.1931	0.7812
	Y	0.9998	<0.001	0.0651	0.0848	0.2604
	Z	0.9986	<0.001	0.0500	0.0813	0.3000
Left Posterior	X	0.9951	<0.001	0.1866	0.1321	0.3906
	Y	0.9995	<0.001	0.1259	0.1044	0.3906
	Z	0.9977	<0.001	0.0700	0.1121	0.3000
Right Anterior	X	0.9835	0.0097	0.3472	0.2001	0.9114
	Y	0.9998	<0.001	0.0564	0.0843	0.2604
	Z	0.9979	<0.001	0.0750	0.0976	0.3000
Right Posterior	X	0.9960	0.0034	0.1910	0.1161	0.3906
	Y	0.9993	<0.001	0.1389	0.1195	0.3906
	Z	0.9974	<0.001	0.1000	0.1161	0.3000

**Fig 7:**
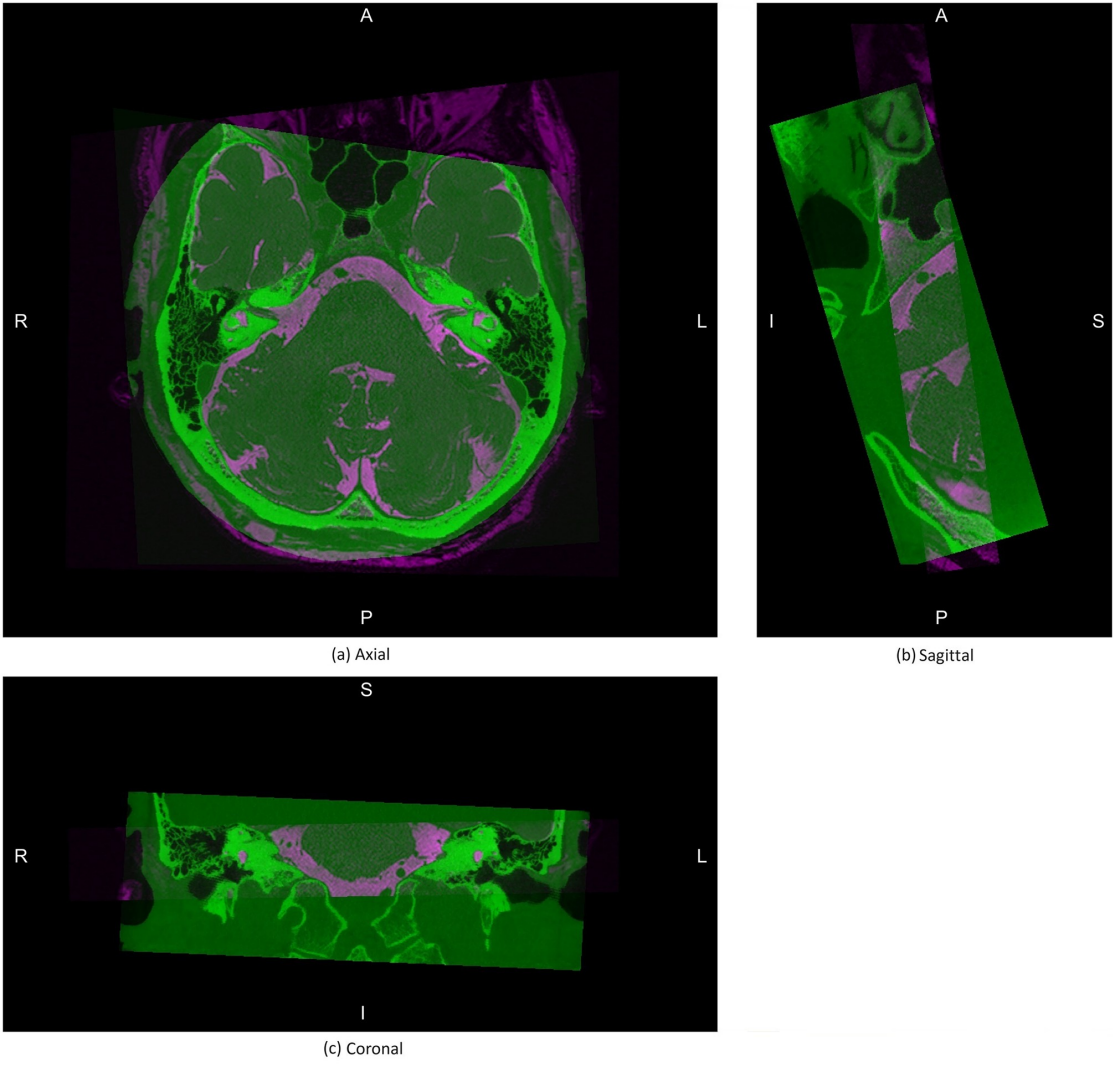
Overlap between the mid slices of the CT and MR images in the proposed coordinate system. A, P, S, I, R, and L indicate anterior, posterior, superior, inferior, right, and left directions respectively.

**Fig 8:**
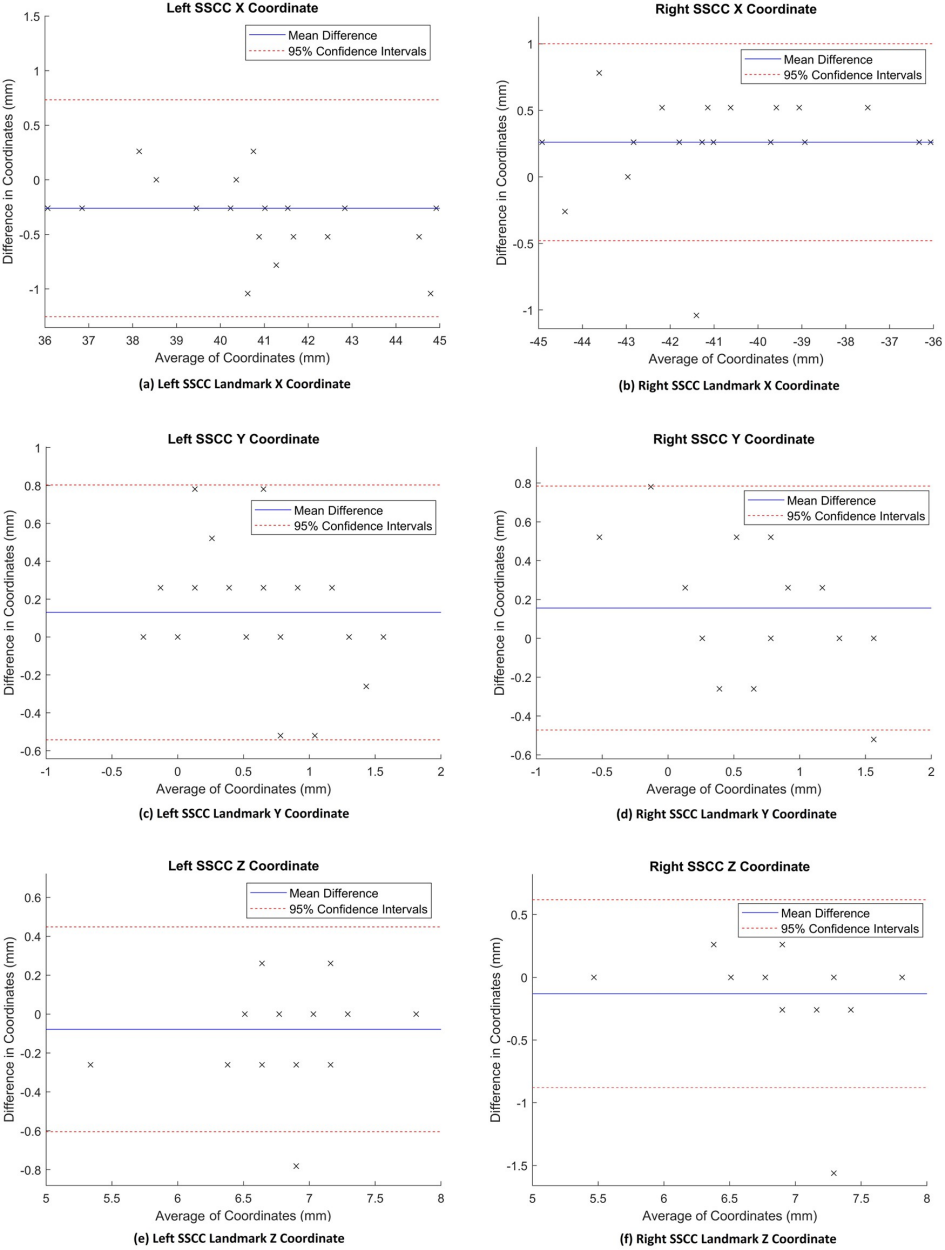
Bland-Altman (difference) plots that compare the CT and MR coordinates after co-registration. The x axis of each plot shows the average coordinate value (X, Y, or Z) of CT and MR landmarks. The y axis shows the error (difference) between the CT and MR coordinate values of a landmark. The solid blue line shows the mean difference and the red dotted lines show the 95% confidence intervals.

Table 5 gives the results of the accuracy analysis. Agreement between CT and MR coordinates after converting to the proposed coordinate system is high, and significant. The distance between points is low as illustrated by the distance metrics in mm. A visual representation of the accuracy is illustrated in the co-registered MR and CT images in Fig 7). Fig 8 shows Bland-Altman (difference) plots that compare the coordinates of each landmark in CT and MR images after they have been co-registered. As can be seen from the figure, the error (distance between the CT and MR points) is low and spread evenly across the scale.

**Table 5:** Target registration error for the SSCC landmarks. The voxel dimension for each of the X, Y, and Z directions is 0.2604mm.

Landmark	Coordinate	r	p	Difference (mm)		
				Mean	Std Dev	Max
Left Superior	X	0.9863	<0.001	0.4427	0.3494	1.3020
	Y	0.8816	<0.001	0.2604	0.2535	0.7812
	Z	0.9201	<0.001	0.1823	0.2087	0.7812
Right Superior	X	0.9915	<0.001	0.3906	0.2314	1.0416
	Y	0.9016	<0.001	0.2604	0.2390	0.7812
	Z	0.8507	<0.001	0.2083	0.3442	1.5624

Table 6 shows the comparison of each selected landmark with its orthogonal projection on the LSCC plane. The high (and significant) agreement rates, as well as the low distances between the selected coordinates and their projections, validate the planarity assumption.

**Table 6:** Comparison of the original landmarks with their orthogonal projections on the best-fit plane. The voxel dimension for each of the X, Y, and Z directions is 0.2604mm.

Modality	Coordinate	r	p	Difference (mm)		
				Mean	Std Dev	Max
CT	X	1.0000	<0.001	0.0023	0.0024	0.0094
	Y	1.0000	<0.001	0.0203	0.0180	0.0764
	Z	0.9998	<0.001	0.0534	0.0496	0.1647
MR	X	1.0000	<0.001	0.0017	0.0016	0.0075
	Y	1.0000	<0.001	0.0185	0.0155	0.0573
	Z	0.9994	<0.001	0.0666	0.0443	0.1682

## Discussion

Multiple factors contribute to the reliability of a coordinate system, including the resolution of the imaging modality, precise definition of the landmark and experience of the user [35, 36]. In this study we were able to identify landmarks which demonstrated significant intra-rater (Tables 1 and 2) and inter-rater reliability (Tables 3 and 4 respectively) thus suggesting that these landmarks are precise and reproducible for both CT and MR. As expected, the intra-rater variability was slightly more accurate than the inter-rater variability [37]. Similarly, as expected, given the larger voxel size of the MR, its reliability was better than that of CT. In addition, this study also showed that the selected landmarks on the LSCCs are planar (or their deviation from planarity is negligible), further supporting the findings of Santina et. al. [15].

Accuracy related to a coordinate system can be considered in the setting of image guided navigation systems or with regards to co-registration of images. In both of these domains, there is no absolute standardised level of accuracy required [38]. In image guided surgical navigation, it is reasonable that accuracy should depend on the complexity and precision that the surgical technique requires. In lateral skull based imaging guidance, the value of 0.5mm has been suggested [39].

Accuracy of a coordinate system with regards to co-registration of images can be performed via the comparison of rigid landmarks, surface volumes or by using segmentation [40, 41]. Each of these methods in themselves have limitations; in the absence of external fiducial systems, rigid landmarks that are visualised on both CT and MR are limited, surface volume is limited by the visualised base of skull on the small field-of-view images and segmentation is time consuming. Although the majority of the literature in this area rely on external fiducial systems, there have been two studies which have investigated the use of non-invasive registration for image guidance [42, 43]. These two studies rely on surface landmark acquisition, which is obtained using CT. While both proved accurate (mean target registration error of 0.51mm and 0.23mm respectively), given the reliance on CT bone imaging for surface landmark acquisition, these methods are not translatable to MR imaging.

The proposed methods in this study have the advantage of requiring only four easily obtained landmarks. This is similar in number to the landmarks used for the Frankfort horizontal plane as well as other similar coordinate systems [2] and vastly less than the number of landmarks that have been assessed for the use in craniofacial skeletal morphology [3].

### Limitations

We assessed the accuracy of the coordinate system in both CT and MR by using a third set of rigid landmarks. This method had the benefit of also proving an accuracy assessment of the subsequent co-registered images. The third set of rigid landmarks (superior SCC) is a relatively subjective landmark, as there is no one specific point to accurately place a marker, and thus is prone to variation in selection particularly in the z and y planes, and this is demonstrated in the results (Section 5). Despite this limitation, the results demonstrate that using the proposed methodology, a coordinate system is obtained, with mean target registration error within the expected limits of suitability for lateral skull base surgery. When comparing the coordinates of the landmarks on CT and MRI after co-registration, the differences between coordinates are greatest in the X axis (right to left) up to 1mm, and y axis (anterior to posterior) up to 0.8mm. This is related to the placement of the landmark along the junction of the SCC and the vestibule, where there is subjectivity in placement abutting the vestibule, particularly in the x and y axis. In the absence of a simple, objective rigid landmark for accuracy assessment, futures studies in the area could compare this coordinate system to one obtained from full brain imaging, however this would need to be assessed on larger field of view images that are obtained contemporary to the smaller field of view images.

While the accuracy of the mean target registration errors was sufficient, it must be noted that the maximum values for both intra-rater reliability and inter-rater reliability exceeded the suggested 0.5mm threshold. On review of the raw data, it was felt that in addition to the aforementioned subjectivity, these errors may have been exacerbated by either transcription error or human error. Both of which cannot be excluded from this study. These discrepancies raise concern for the use of manual generated landmarks in image guided surgery, particularly in the temporal bone where there are few reproducible landmarks seen on both CT and MRI. While the overall aim would be to develop a coordinate system reliant on automatically generated landmarks, as yet, the literature in this field suggest sub-optimal accuracy data as compared to the results in the literature of manually acquired landmarks [39, 44, 45]. The methods used in this study are reliant on the availability of experienced users acquiring the landmarks as the use of an experienced user has previously been identified as an important factor influencing the reproducibility of landmark identification [35]. Another limitation is the limited sample size of three experts who selected landmarks, however this number is on par with other similar studies [3, 8]. A future avenue of research would be to develop an automatic system for landmark detection that not only reduces the reliance on experts but is also comparable in accuracy.

We propose that this system has the potential to provide an accurate coordinate system in the setting of pathology / craniofacial asymmetry given the limited effect of craniofacial malformations on the orientation of the lateral semicircular canals [8, 20]. However, in the present study we assessed only 20 patients (40 CT and MR images in total), demonstrating no cochlear or temporal bone pathology. Thus, accuracy of this method in known pathology or craniofacial malformation has not been assessed but is proposed to be investigated in future work.

### Future applications

The ability of a coordinate system for accurate co-registration, both of chronologically separate scans as well as different imaging modalities, leads to several useful applications. For example, it would allow for the localisation of any given object using an atlas based approach for segmentation or surgical planning [46, 47]. Accurate definition of the sagittal midline plane, obtained from such a coordinate system, can allow symmetry extraction wherein two hemispheres can be compared for pathology. Additionally, in the field of cochlear implant surgery, this method could streamline the assessment of post operative electrode position where post operative MRI is often limited by image artefact or safety restrictions of the implanted device. This would expand on the work by Dragovic et al [48] where the co-registered images could be used for an accurate trajectory mapping of an electrode using the preoperative MRI which would demonstrate the positioning of the scala tympani and scala vestibuli, as well as the post operative CT demonstrating the electrode position. In addition, the coordinate system could be used as a part of a surgical planning pathway, where electrode vectors are calculated with specific cochlear implant electrode dimensions to guide a personalised surgical plan for cochlear implantation. Further research would investigate the benefit of this planning pathway on surgical outcomes in cochlear implant surgery, particularly the meaningful benefit of hearing preservation [49].

## Conclusion

This study presented a practical and accurate method of defining a coordinate system which is applicable to the high resolution / small field of view images of the temporal bone region acquired by both CT and MR. We showed using CT and MR images of 20 patients that the method is reproducible and accurate. The localised landmarks enable this coordinate system to be used in cases where common landmarks used in existing coordinate systems defined for the head are not available. The multi-modal nature of the coordinate system makes it possible to be used in downstream tasks such as image co-registration, development of atlas-based methods (such as for segmentation) and disease diagnosis.

## Supporting information

S1 Appendix Coordinate selection data. (XLSX)
